# Neuronal Excitatory State Is Linked to Stress Resilience

**DOI:** 10.1093/geroni/igab046.1438

**Published:** 2021-12-17

**Authors:** Joseph Zullo, Bruce Yankner

**Affiliations:** Harvard Medical School, Boston, Massachusetts, United States

## Abstract

The aging human brain is a study in both the importance and limitations of human stress response factors. Individual neurons can maintain functionality for 80 or more years, testifying to the potency of their stress response pathways. However, failure of these pathways during aging drastically increases the risk of neurodegenerative diseases. The transcriptional repressor REST is induced in the brains of long-lived humans but is lost in neurodegenerative disease. Here, we explore one modality of REST’s protective effects: regulation of neuronal excitability. We show that excitatory capacity and stress response are inversely correlated in the human brain. We find that REST and its C. elegans orthologs repress neuronal excitation in response to stressful conditions. Further, exogenously suppressing neuronal excitation restores stress resistance to REST-deficient animals, while enhancing stress response in wildtype ones. Thus, regulation of neuronal activity is an important aspect of neuronal stress response and a potential therapeutic modality.

